# Supplementary material for: The chloroplast 2-cysteine peroxiredoxin functions as thioredoxin oxidase in redox regulation of chloroplast metabolism
Source: eLife. 2018 Oct 12;7:e38194. doi: 10.7554/eLife.38194 (PMC6221545; doi:10.7554/eLife.38194)
Supplement: Figure 2—source data 1. [file elife-38194-fig2-data1.docx]

**Figure 2 – Source data. Values from the FBPase activity test** (200 and 500μM DTT) (Figure 2B and 2D) (n=3). Experiments were performed with previously frozen aliquots from the same stroma extract on different days of analysis as described in the legend to Figure 2. Exp: experiment; FBPase: fructose-1,6-bisphosphatase; ox: oxidized; SD: standard deviation of the mean (n-1); Str: Stroma; Trx: thioredoxin.

| FBPase (200 µM DTT)  [nmol/mg^.^min] | Exp 1 | Exp 2 | Exp 3 | mean | SD |
| --- | --- | --- | --- | --- | --- |
| Str | 61.9 | 55.7 | 61.9 | 59.8 | 3.5 |
| Str + Trx-f1 | 79.4 | 88.9 | 84.9 | 84.4 | 4.7 |
| Str + 2-CysPrx A ox | 73.1 | 65.8 | 74.8 | 71.3 | 4.8 |
| Str + 2-CysPrx A ox + Trx-f1 | 14.9 | 12.6 | 16.1 | 14.6 | 1.7 |

| FBPase (500 µM DTT)  [nmol/mg^.^min] | Exp 1 | Exp 2 | Exp 3 | mean | SD |
| --- | --- | --- | --- | --- | --- |
| Str | 119.4 | 121.9 | 113.2 | 118.2 | 4.4 |
| Str + Trx-f1 | 136.0 | 108.6 | 138.7 | 127.8 | 16.6 |
| Str + 2-CysPrx A_ox_ | 108.6 | 116.2 | 110.4 | 111.7 | 3.9 |
| Str + 2-CysPrx A_ox_ + Trxf-1 | 74.7 | 70.9 | 52.1 | 65.9 | 12.1 |
| Str + 2-CysPrx_red_ | 126.6 | 146.7 | 140.7 | 138.0 | 10.3 |
