# Supplementary material for: The chloroplast 2-cysteine peroxiredoxin functions as thioredoxin oxidase in redox regulation of chloroplast metabolism
Source: eLife. 2018 Oct 12;7:e38194. doi: 10.7554/eLife.38194 (PMC6221545; doi:10.7554/eLife.38194)
Supplement: Figure 3—source data 1. [file elife-38194-fig3-data1.docx]

**Figure 3 – Source data.** FBPase activity in the presence of different Trx-f1 concentrations. Experiments were performed with aliquots from two different stroma extracts on different days of analysis. Exp: experiment; FBPase: fructose-1,6-bisphosphatase; ox: oxidized; SD: standard deviation of the mean (n-1); Str: Stroma; Trx: thioredoxin.

| Trx-f1 dependency [nmol/mg^.^min] | Exp 1 | Exp 2 | Exp 3 | mean | SD |
| --- | --- | --- | --- | --- | --- |
| Control stroma | 42.2 | 45.5 | 49.8 | 45.8 | 3.8 |
| Trx-f1 0.625 µM +2-CysPrxA 5 µM | 34.7 | 39.0 | 36.4 | 36.7 | 3.1 |
| Trx-f1 1.25 µM +2-CysPrxA 5 µM | 30.2 | 30.9 | 32.3 | 31.1 | 1.1 |
| Trx-f1 2.5 µM +2-CysPrxA 5 µM | 20.9 | 25.3 | 26.9 | 24.4 | 2.2 |
| Trx-f1 5 µM +2-CysPrxA 5 µM | 17.7 | 16.6 | 20.1 | 18.1 | 2.4 |
