# Supplementary material for: The chloroplast 2-cysteine peroxiredoxin functions as thioredoxin oxidase in redox regulation of chloroplast metabolism
Source: eLife. 2018 Oct 12;7:e38194. doi: 10.7554/eLife.38194 (PMC6221545; doi:10.7554/eLife.38194)
Supplement: Figure 4—source data 1. [file elife-38194-fig4-data1.docx]

**Figure 4 – Source data.** Values of the MDH activity test shown in figure 4B. Experiments were performed with aliquots from three different stroma extracts. The experiment-specific background was subtracted from each value. Exp: experiment; MDH: malate dehydrogenase; ox: oxidized; SD: standard deviation of the mean (n-1); Str: Stroma; Trx: thioredoxin. The background was subtracted and the result normalized (^n^). *The % of MDH represents a mean calculated from the % of normalized value of each experiment. The letters above the columns designate groups of statistically significant difference (p<0.05), according to post hoc Tukey’s HSD.

| Δ 10^-3^ Abs/min | Exp 1 | Exp 2 | Exp 3 | Exp 4 | *% of MDH activation^n^ | ±SD^n^ | |
| --- | --- | --- | --- | --- | --- | --- | --- |
| DTT | -2.30 | -4.70 | -5.55 | -5.00 | 100.0^a^ | 0.0 |  |
| Trx-m1 + DTT |  | -4.27 | -6.11 | -3.89 | 92.9^a^ | 13.3 |  |
| Trx-m4 + 2-CysPrx_ox_ | -1.90 | -3.53 | -2.79 |  | 69.3^ab^ | 13.7 |  |
| Trx-x + 2-CysPrx_ox_ |  | -1.78 | -2.30 | -2.00 | 39.7^bc^ | 1.5 |  |
| Trx-f1 + 2-CysPrx_ox_ | -1.11 | -1.55 | -1.47 |  | 35.8^c^ | 9.0 |  |
| CDSP32 + 2-CysPrx_ox_ | -0.87 | -0.33 | -0.87 |  | 20.1^cd^ | 12.8 |  |
| Trx-m1 + 2-CysPrx_ox_ | -0.19 | 0.28 | -0.24 |  | 2.2^d^ | 2.3 |  |
