# Supplementary material for: The chloroplast 2-cysteine peroxiredoxin functions as thioredoxin oxidase in redox regulation of chloroplast metabolism
Source: eLife. 2018 Oct 12;7:e38194. doi: 10.7554/eLife.38194 (PMC6221545; doi:10.7554/eLife.38194)
Supplement: Figure 5—source data 1. [file elife-38194-fig5-data1.docx]

**Figure 5 – Source data. Changes in MDH and PRK activities in WT, *2cysprxAB* mutants and complemented lines C1 and C2 after light-dark transition.** Four week old *A. thaliana* WT plants, *2cysprxAB* mutants, C1 and C2 lines were illuminated for 30 min with 650 µmol quanta m^-2^ s^-1^ in order to activate the chloroplast enzymes NADPH-MDH and PRK. Leaves were then darkened for indicated time periods and immediately frozen in liquid nitrogen. The NAD(P)H-oxidation was followed at 340 nm. Each experiment (n) was conducted with different sets of plants. (A) Initial MDH activities for the time points 0 s and 10 s of WT and *2cysprxAB* leaf extracts with n = 5-6 and n = 3 for C1 and C2 lines. (B) Percent inactivation was determined for each genotype by calculating the ratio of the activities at 10 and 0 s. (C) Ratio of initial vs. total PRK-activity after light-dark transition in WT, *2cysprxAB*, C1 and C2 leaf extracts. The PRK activation state was significantly higher in *2cysprxAB* than in all other lines after 300 s of darkening (n=6; p<0.005 for WT, n=3 and p<0.01 for both C1 and C2 (one-way ANOVA with post-hoc Tukey HSD test).

A)

|  |  | Initial MDH activity [nmol NADPH/ min mg protein] | | | | | | | | | |
| --- | --- | --- | --- | --- | --- | --- | --- | --- | --- | --- | --- |
| Genotype | | | Exp 1 | Exp 2 | Exp 3 | Exp 4 | Exp 5 | Exp 6 | Mean | ± SE |  |
| WT 0 s | | | 28.1 | 23.9 | 12.6 | 12.0 | 8.3 | 18.4 | 17.2 | 2.9 |  |
| *2cysprxAB* 0 s | | | 20.2 | 13.4 | 10.7 | 27.1 | 9.8 | 17.9 | 16.5 | 2.5 |  |
| WT 10 s | | | 14.2 | 5.2 | 9.0 | 8.1 | 8.3 | 12.9 | 9.6 | 1.2 |  |
| *2cysprxAB* 10 s | | | 16.3 | 13.5 | - | 13.7 | 14.8 | 16.6 | 15.0 | 0.6 |  |

| Genotype | Exp 1 | Exp 2 | Exp 3 | mean | SE |
| --- | --- | --- | --- | --- | --- |
| C1 0 s | 18.8 | 22 | 22.1 | 21.0 | 0.9 |
| C1 10 s | 16.6 | 9.7 | 12 | 12.8 | 1.7 |
| C2 0 s | 21.9 | 26.1 | 30.4 | 26.1 | 2.0 |
| C2 10 s | 25.4 | 15.3 | 16.5 | 19.1 | 2.6 |

(B)

| MDH activity  after 10 s darkening in % of 0 s | | |
| --- | --- | --- |
| Genotype |  |  |
| WT 10 s | 55.8 |  |
| *2cysprxAB* 10 s | 90.9 |  |
| C1 10 s | 61.0 |  |
| C2 10 s | 73.2 |  |

(C)

| Initial/total PRK activity [nmol NADH/ min mg protein] | | | | | | | | |
| --- | --- | --- | --- | --- | --- | --- | --- | --- |
|  | Exp 1 | Exp 2 | Exp 3 | Exp 4 | Exp 5 | Exp 6 | Mean | ± SD |
| WT 300 s | 0.2 | 0.5 | 0.5 | 0.3 | 0.3 | 0.5 | 0.4 | 0.1 |
| *2cysprxAB* 300 s | 1.7 | 1.4 | 0.8 | 1.0 | 1.0 | 1.1 | 1.2 | 0.3 |
| C1 300 s | 0.2 | 0.4 | 0.4 | - | - | - | 0.3 | 0.1 |
| C2 300 s | 0.3 | 0.4 | 0.5 | - | - | - | 0.4 | 0.1 |
