# Supplementary material for: The chloroplast 2-cysteine peroxiredoxin functions as thioredoxin oxidase in redox regulation of chloroplast metabolism
Source: eLife. 2018 Oct 12;7:e38194. doi: 10.7554/eLife.38194 (PMC6221545; doi:10.7554/eLife.38194)
Supplement: Figure 7—source data 1. [file elife-38194-fig7-data1.docx]

**Figure 7 – Source data.** Fresh weight of WT and *2cysprxAB* plants as well as complemented lines C1 and C2 after growth in fluctuating light for three weeks. The fluctuating light consisted of 40s L/5s L (left) and 80s L/10s L (right) as described in the methods part. Statistics for fresh weight included n=13 plants from 3 independent cultivations and was calculated with one-sided ANOVA followed by Tukey’s HSD. Growth of the *2cysprxAB* mutant was significantly lower than growth of WT and C1 and C2 with p<0.01.

| Growth as fresh weight [mg] | | |  |  |  |  |  |  |  |
| --- | --- | --- | --- | --- | --- | --- | --- | --- | --- |
| Fluctuating light (40s L/5s L) | | |  |  | Fluctuating light (80s L/10s L) | | |  |  |
| WT | *2cysprxAB* | C1 | C2 |  | WT | *2cysprxAB* | C1 | C2 |  |
| 17.5 | 12.4 | 17.50 | 15.30 |  | 13.40 | 18.50 | 16.80 | 14.9 |  |
| 15.3 | 11.4 | 20.60 | 13.60 |  | 16.70 | 12.50 | 15.20 | 13.2 |  |
| 12.6 | 10.6 | 20.30 | 15.80 |  | 12.40 | 16.40 | 13.20 | 10.5 |  |
| 16.4 | 14.8 | 15.60 | 16.70 |  | 15.80 | 17.80 | 11.50 | 14.6 |  |
| 14.2 | 11.9 | 13.50 | 15.50 |  | 12.90 | 17.50 | 17.00 | 12 |  |
| 19.6 | 12.5 | 14.60 | 14.20 |  | 11.50 | 19.30 | 9.50 | 13.6 |  |
| 15.3 | 12.4 | 17.60 | 16.70 |  | 12.10 | 14.20 | 12.40 | 11.2 |  |
| 17.5 | 14.2 | 15.80 | 18.40 |  | 17.20 | 19.10 | 12.30 | 9.6 |  |
| 14.2 | 10.4 | 17.40 | 12.50 |  | 11.50 | 15.20 | 13.90 | 15.4 |  |
| 15.3 | 12.5 | 15.90 | 14.40 |  | 12.40 | 14.30 | 11.80 | 9 |  |
| 16.00 | 12.30 | 16.80 | 16.80 |  | 13.50 | 17.50 | 12.50 | 13.1 |  |
| 21.40 | 13.60 | 14.30 | 16.50 |  | 16.30 | 16.20 | 9.50 | 13.6 |  |
| 20.70 | 13.90 | 12.70 | 15.40 |  | 12.10 | 15.20 | 15.60 | 10.9 |  |
|  |  |  |  |  |  |  |  |  |  |
| 16.62 | 12.53 | 16.35 | 15.52 |  | 13.68 | 16.44 | 13.17 | 12.43 | mean |
| 2.64 | 1.32 | 2.37 | 1.57 |  | 2.07 | 2.08 | 2.45 | 2.06 | ±SD |
