# Supplementary material for: The chloroplast 2-cysteine peroxiredoxin functions as thioredoxin oxidase in redox regulation of chloroplast metabolism
Source: eLife. 2018 Oct 12;7:e38194. doi: 10.7554/eLife.38194 (PMC6221545; doi:10.7554/eLife.38194)
Supplement: Supplementary file 1. — for: forward primer, rev: reverse primer. [file elife-38194-supp1.docx]

**Supplementary Table 1. Primers used for cloning.** for: forward primer, rev: reverse primer.

| **Name** | **Nucleotide sequence** |
| --- | --- |
| **AtTrxF1for**  **AtTrxF1rev**  **AtTrxM1for**  **AtTrxM1rev**  **AtTrxM4for**  **AtTrxM4rev**  **AtTrxXfor**  **AtTrxXrev**  **AtCDSP32for**  **AtCDSP32rev**  **At2-CysPrxAfor**  **At2-CysPrxArev**  **GK-RBfor**  **At2CPCDS-NcoIrev**  **At2CPCDS-XhoIfor**  **At2CPprm-XhoIrev**  **At2CPprm-EcoRIfor**  **At2-CysPrxgenF**  **At2-CysPrxgenR**  **AtFBPasefor**  **AtFBPaserev** | **5´ATATACATATGAGCTTAGAAACCGTTAATGTCAGTG 3´**  **5´ATATAGGATCCTCATCCGGAAGCAGCAGAC 3´**  **5´ATATACATATGGAAGCTCAGGACACTGCTAC 3´**  **5´ATATAGGATCCTTACAAGAATTTGTTGATGCTGG 3´**  **5´ATATACATATGGAGGCTCAGGACACCACTG 3´**  **5´ATATAGGATCCTTACTCGACCAAGAATCTTTC 3´**  **5´ATATACATATGGGCGGAATCAAAGAGATTGG 3´**  **5´ATATAGGATCCTTAAGCAACAGATATTGAGTTCAAG 3´**  **5´ATATACATATGGCTGGAGCGGCGTCTCC 3´**  **5´ATATAGGATCCTTAATAAGTGACACGAACGCC 3´**  **5´ATATACATATGGCCCAGGCCGATGATCTTCC 3´**  **5´ATATAGAATTCTAAATAGCTGAGAAGTACTC 3´**  **5´ATATCATCGTGGAAAAAGAAGACG 3´**  **5´AAAAGGTACCCTAAATAGCTGAGAAGTACTCT 3´**  **5´AAAAACTCGAGATGGCGTCTGTTGCTTCTTCAA 3´**  **5´AAAAGAATTCACTAGTATATACTACGTG 3´**  **5´AAAACTCGAGTGCTGCATCCCTTG 3´**  **5´ATATGGATGATCTTCCACTGGTTGG 3´**  **5´ATAT**GGAGGGTTCTCATTGTCTCATC **3´**  5’CCCCCCCATATGGGATACGAACTTCAAACGTTGACGGGC 3´  5’CCCCGGATCCTCAAGCCAAGTACTTCTCCAGCTTCTC 3´ |
